# Ongoing Implementation and Prospective Validation of Artificial Intelligence/Machine Learning Tools at an African Drug Discovery Center

**DOI:** 10.1021/acsmedchemlett.4c00243

**Published:** 2024-06-21

**Authors:** Jason Hlozek, Kelly Chibale, John G. Woodland

**Affiliations:** † Department of Chemistry and Holistic Drug Discovery and Development (H3D) Center, University of Cape Town, Cape Town 7701, South Africa; ‡ South African Medical Research Council Drug Discovery and Development Research Unit, Institute of Infectious Disease and Molecular Medicine, University of Cape Town, Cape Town 7925, South Africa

## Abstract

Artificial intelligence (AI) and machine learning (ML) are anticipated to accelerate drug discovery programs. Following our development of an end-to-end virtual screening cascade at the University of Cape Town (UCT) Holistic Drug Discovery and Development (H3D) Center, we report the ongoing implementation of open-source AI/ML tools for use in resource-constrained settings.

Developing novel medicines is a lengthy and challenging process. Candidate molecules need to advance through the various stages of the drug discovery pipeline, from hit discovery and medicinal chemistry optimization to clinical trials and ultimately (if successful) regulatory approval. During the early stages of this process, the physicochemical and biological properties of a chemical compound need to be optimized to discover a potential treatment that is both safe and efficacious. However, high attrition rates at all stages of the drug discovery value chain result in lengthy timelines and exorbitant costs–with current estimates of more than 10 years and over $1 billion, respectively.^1^ This significant investment–without the guarantee of success–is often prohibitive for drug discovery research for the treatment of infectious diseases that disproportionately affect populations in low- and middle-income countries (LMICs).

With the advent of the fourth industrial revolution and advances in artificial intelligence (AI) and machine learning (ML) technologies, much hope has been placed by the scientific community in data science tools to reduce attrition rates and accelerate drug discovery programs.^2^ These tools could offer especial value to resource-constrained settings such as those in LMICs. One such example is the University of Cape Town (UCT) Holistic Drug Discovery and Development (H3D) Center,^3^ which initiated the adoption of AI/ML tools in its research programs in 2021 through a collaboration with the Ersilia Open Source Initiative,^4^ a technology nonprofit that focuses on the dissemination of data science tools in low-resource settings.

This productive collaboration yielded the development of the ZairaChem pipeline, an automated AI/ML model training tool which produces state-of-the-art QSAR/QSPR models across a variety of chemical and biological assays. This pipeline implementation was intentionally selected as a sustainable way to facilitate the ongoing development of predictive AI/ML models for user-specific regions of chemical space while automating the often-laborious trial-and-error process of model optimization. This approach allows for new models to be trained when additional experimental assays are brought online or are identified as critical for driving a project. In addition, existing AI/ML models can easily be retrained to benefit from new experimental data whereas static AI/ML models would, on the other hand, quickly become outdated.

The development of ZairaChem and the resulting implementation at H3D, which included both retrospective and prospective validation as well as benchmarking against external data sets, was subsequently shared with the broader scientific community.^5^ In this Viewpoint, we report further prospective validation over the 2023 period and discuss the practical aspects of the ongoing implementation of these AI/ML models, which could serve as an example and inspiration for others at nascent drug discovery centers worldwide and particularly in LMICs.

## Model Implementation and Prospective Validation at the H3D Center

The original set of AI/ML models was selected to predict the experimental outcome of key assays that are frequently used for decision making in screening cascades for drug discovery programs targeting *Plasmodium falciparum* and *Mycobacterium tuberculosis*, the causative agents of malaria and tuberculosis (TB), respectively. Typically, medicinal chemists design dozens of potential compounds but have limited resources for chemical synthesis; hence, the suite of models trained through ZairaChem was assembled into a virtual screening cascade to facilitate their use by medicinal chemists to prioritize those compounds for synthesis that are more likely to possess desirable druglike properties.

Our virtual screening cascade is intended to be used in conjunction with existing software to generate complementary data points to facilitate decision-making. Considering there is no substantial limit on the number of compounds that can be run through the cascade, chemists are encouraged to “think big” and examine much longer lists of compounds than they might otherwise propose, allowing them to cast the net more widely and so interrogate a broader chemical space. Furthermore, as many of these models have been trained on in-house data for malaria and TB programs, they are potentially more relevant to other researchers in LMICs than more general models available online, such as the MAIP webtool.^6^

**Figure 1:**
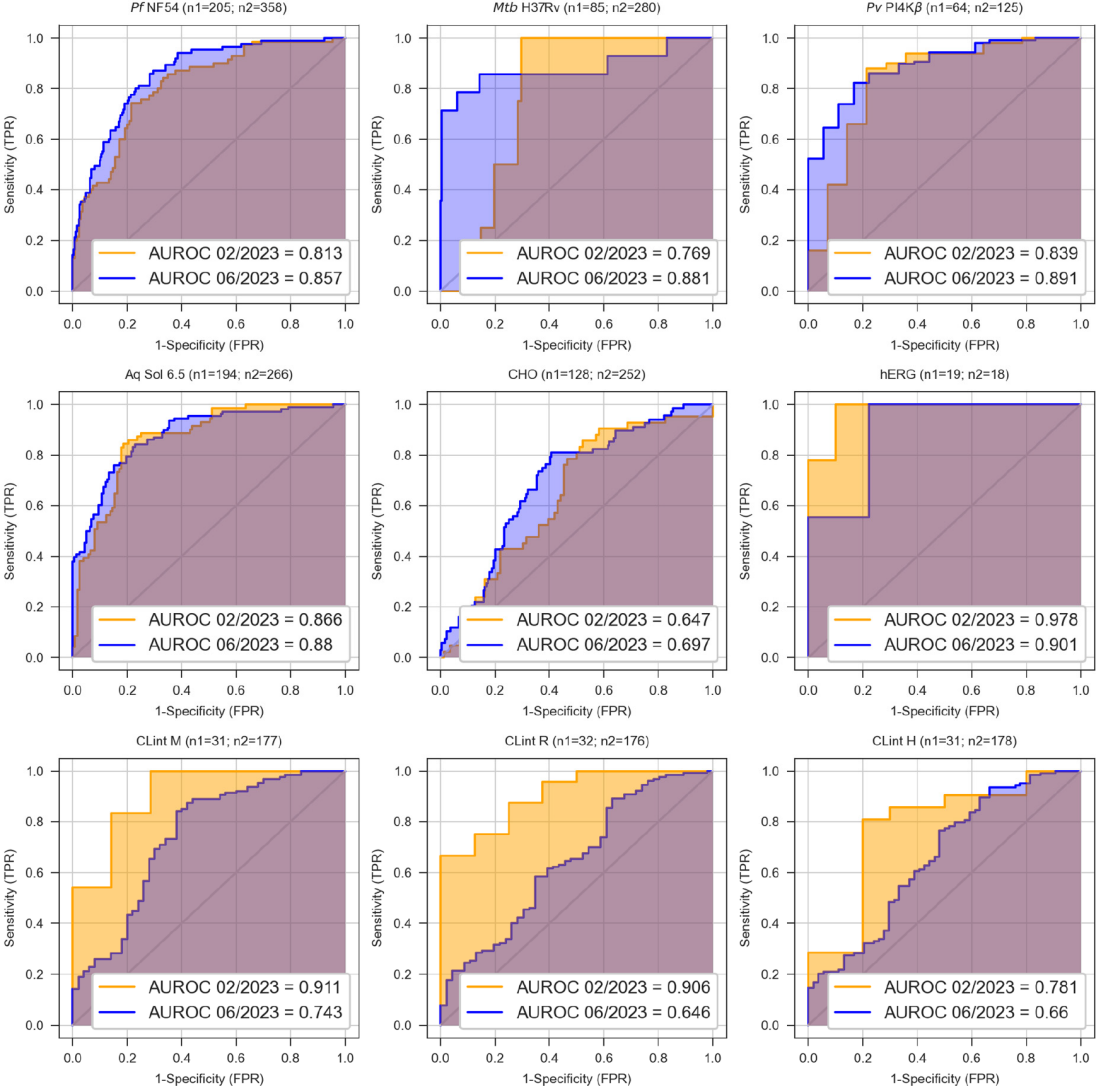
Prospective model performance for the virtual screening cascade AI/ML models at H3D in 2023 which showed excellent agreement of the model predictions with experimental data. ROC curves are plotted for the prospective data generated in the months following the training of models on in-house data up to February 2023 (orange) and June 2023 (blue), respectively.

Our models undergo biannual retraining to continuously incorporate the most recent in-house data to maximize model applicability for current research programs. Both sets of models in 2023, trained on H3D’s in-house data up to February and June of that year, respectively, showed good prospective performance for experimental data generated over the following months. In particular, the whole-cell pathogen activity and aqueous solubility models, with the largest volume of training data, showed consistently high performance metrics, with an area under the receiver operating characteristic (AUROC) curve of between 0.77 and 0.89 (Figure 1). The microsomal intrinsic clearance (CL_int_) models dropped in performance in the second half of 2023 due to a large batch of compounds from two chemical series that are poorly represented in the training set, indicating the need for model retraining to improve performance in that particular chemical space.

## Development of New Models

**Figure 2:**
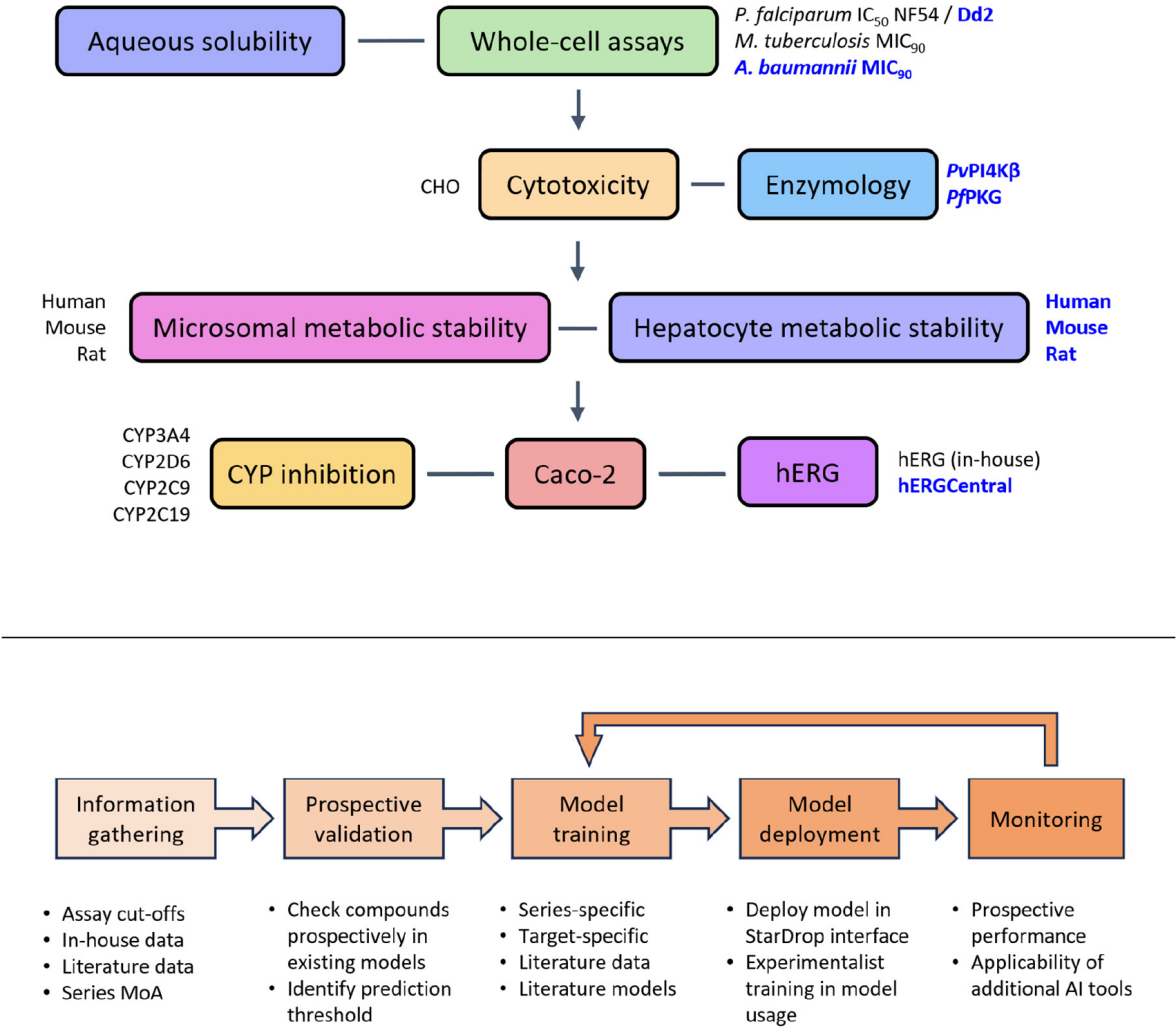
Current models comprising the H3D virtual screening cascade (top) in which newly developed models are emphasized in blue. Illustration of the typical workflow (bottom) for the development of new models to be made available for screening by experimentalists.

Since the adoption of these AI/ML models by drug discovery teams at the H3D Center, additional data sets and areas of need have been identified for model development (Figure 2). These include predictions for the inhibition of the *P. vivax* phosphatidylinositol 4-kinase beta (*Pv*PI4Kβ) enzyme, the *P. falciparum* cyclic GMP-dependent protein kinase (*Pf*PKG), and the inhibition of the formation of β-hematin (the synthetic equivalent of malaria pigment), which represent three important drug targets in antimalarial drug discovery research. These ZairaChem models show excellent predictive performance with AUROC scores above 0.89 (data not shown) likely owing, in part, to the focused nature of the prediction task without the presence of confounding factors in whole-cell assays such as permeability or the presence of transport proteins. In addition, a second model to predict activity against the human ether-á-go-go-related-gene (hERG) ion channel, an important indicator of cardiotoxicity, has been developed from the hERGCentral database^7^ to expand the chemical space of the training set.

Not only can new models be developed for the early hit-to-lead phase of existing projects to prioritize compounds for synthesis, but these tools can also be used to screen large compound libraries to discover new chemical matter as starting points for drug discovery projects. For this purpose, we have pursued the development of more general activity prediction models that can facilitate virtual screening for new chemical hits against *P. falciparum* and *A. baumannii*, the latter being a member of the ESKAPE pathogens with increasing reports of antimicrobial resistance (AMR) and a bacterium of particular importance at the H3D Center. These models have been trained on large open-access data sets (for example, a curated set of 35 000 compounds from ChEMBL and CO-ADD),^8,9^ exemplifying the versatility of the ZairaChem tool to produce models for a variety of prediction tasks.

## Impediments to the Adoption of AI/ML Tools

As “wet-lab” experimentalists seldom have formal computer science training, the technical complexity and black-box nature of AI/ML is often intimidating and may impede the adoption of data science tools. Furthermore, the computational field is filled with technical concepts that are common knowledge for data scientists but may be unfamiliar to experimental scientists, which may further hinder implementation of these models. Third, many AI/ML pipelines are solely implemented for Linux-based environments through a command-line terminal. Indeed, although the use of the ZairaChem pipeline requires just the “fit” and “predict” commands in a Linux terminal, tools that lack a graphical user interface (GUI) are perceived as inaccessible to many experimentalists. Therefore, a successful strategy to promote the local adoption of AI/ML tools needed to address both a skills-development component, to familiarize experimentalists with key concepts, and a technical component, making ZairaChem as easily accessible as possible.

## Workflow of AI/ML Implementation

A typical workflow for deploying AI/ML models in a drug discovery project at H3D comprises the following steps:

1. An initial project meeting is held with the scientists on the project, most of whom may be medicinal chemists, to discuss factors such as the key assay decision/cutoff values, the available in-house data, potential confounding factors in the data set (e.g., duplicate measurements), knowledge of the mechanism of action (MoA) of the chemical series, and the availability of any relevant literature data.
2. The available in-house data are passed, in a prospective manner, through the two previous iterations of the virtual screening models to estimate the potential accuracy one can expect for further prospective predictions and, therefore, build confidence in the predictions.
3. If the existing data are not well-predicted or the virtual screening cascade models do not have suitable cutoffs for the project in question, a series of new ZairaChem models are trained to identify the most effective manner for modeling the specific chemical space under investigation. This may include training a series-specific model if enough data are available, a target-specific model if the MoA is known, a model that may include any available literature data from other research groups, or utilizing models from the literature via the Ersilia Model Hub.^10^ The modeling data are typically split into training (80%) and test (20%) sets chronologically, with the goal of having the test set capture the most recent project direction.
4. The modeling results are then shared with the experimental scientists in a follow-up meeting and the best-performing model is employed for screening proposed chemical analogs.
5. The models are monitored for ongoing performance through the subsequent design-make-test cycles of chemical optimization, which could trigger model retraining if performance begins to drop or if approximately 30 or more new compounds have been synthesized.

## Key Learnings from Our Experience at the H3D Center

As a very first step to encourage our scientists to implement these predictive AI/ML tools in their projects, an internal webinar was held to introduce scientists to the models in order to demystify these new tools. Careful explanations were given to describe the potential benefits of the models and to elucidate how they were trained and how to interpret the results. Thereafter, “champion scientists” in each disease area under investigation at the H3D Center helped to promote the tools among project teams and also brought ideas and suggestions back to the development team. Continuous communication through project meetings and training sessions provided a forum in which pertinent AI/ML concepts could be introduced or refreshed. Recurring areas of confusion include:

- the difference between “classification” and “regression” models;
- the interpretation of model prediction scores (such as the selection of appropriate thresholds for classification scores);
- model performance metrics such as the AUROC curve;
- and the applicability of a model based on the similarity of the training data compared to the chemical space of interest for predictions.

The use of boxplots to visualize model test-set predictions is particularly helpful in aiding teams to decide on appropriate prediction score thresholds to differentiate classification scores between predicted actives and inactives.

Initially, the process of obtaining model predictions required manual intervention by a data scientist; however, progressing to a GUI-based manner of accessing ZairaChem models has also been an important contributor to lowering the barrier of AI/ML model adoption. Because medicinal chemists at H3D already use the proprietary software package StarDrop to organize project data and because this software package also offers a Python-based plug-in point for AI/ML models, it is currently leveraged for GUI-based, on-demand model predictions. Medicinal chemists and other experimentalists can load a list of compounds and click the checkboxes corresponding to any number of AI/ML models for which they wish to obtain predictions. A custom Python script runs the list of SMILES strings through the corresponding ZairaChem models and returns a list of predictions to be displayed within the standard StarDrop GUI. While far from a sophisticated and flexible model deployment framework, this implementation has been extremely useful in the interim by making the AI/ML models easily accessible within an already-familiar tool.

## Sustainable Capacity Building

An overarching goal of the H3D-Ersilia collaboration is to build data science capacity at an African drug discovery center through the sustainable implementation of AI/ML tools and related skills development. After an initial on-site training period with Ersilia in AI/ML model development, the ongoing implementation of AI/ML models described in this article has been accomplished without the need for ongoing technical support. Rather than static AI/ML assets that will increasingly become out of date and require a trial-and-error approach to train anew, the highly automated ZairaChem tool facilitates continuous development of new models and easy retraining of existing models. Even where data science expertise is available in-house, the automated nature of the pipeline frees the data scientist to pursue other research tasks while still obtaining high-quality models. The pipeline does not require extensive technical expertise nor extensive infrastructure to leverage, which is a crucial factor for resource-constrained research institutions such as many of those in LMICs.

The ZairaChem code is fully open source (https://github.com/ersilia-os/zaira-chem), and the lightweight versions of the virtual screening cascade models are freely available online (https://bit.ly/h3d-app). Taken together, this collaboration represents a landmark success for the transfer of sustainable data science expertise to the nascent African drug discovery domain. H3D and Ersilia aim to continue to build capacity for AI/ML tools at additional centers of African drug discovery research and development by continuing to facilitate data science workshops for African-based researchers and students. In particular, the scientists of the drug discovery cohorts of the Grand Challenges Africa initiative were invited to attend week-long training events hosted by the H3D Foundation and Ersilia, and a session on data science tools is also organized when these scientists conduct on-site visits at the H3D Center. The resources from previous training initiatives are also freely accessible to the scientific community (https://ersilia.gitbook.io/event-fund).

## Future Directions

Following the initial successful deployment of data science tools at H3D, we have begun a concerted effort to improve the usability of the models by increasing the interpretability of model predictions and optimizing models to increase the speed of predictions. We have also begun leveraging the virtual screening cascade models to guide generative AI/ML method algorithms which propose new chemical analogs by searching through chemical space while optimizing for higher model prediction scores.

Recently, the selection of Dr. Kelly Chibale as a Schmidt Futures AI2050 Senior Fellow has funded a project that couples AI/ML predictions to physiologically-based pharmacokinetic (PBPK) modeling with the goal to tailor drug dosages for African patient populations.^11^ In addition, the project envisages developing open-source AI/ML models and significant capacity building for data science tools in Africa. With this work, we are excitedly moving toward a cloud-based deployment of AI/ML models that will facilitate intuitive and scalable access to data science tools to democratize AI/ML technology for drug discovery in Africa.
